# Association of FTO Mutations with Risk and Survival of Breast Cancer in a Chinese Population

**DOI:** 10.1155/2015/101032

**Published:** 2015-06-04

**Authors:** Xianxu Zeng, Zhenying Ban, Jing Cao, Wei Zhang, Tianjiao Chu, Dongmei Lei, Yanmin Du

**Affiliations:** Department of Pathology, The Third Affiliated Hospital of Zhengzhou University, Zhengzhou 450052, China

## Abstract

Recently, several studies have reported associations between fat mass and obesity-associated (FTO) gene mutations and cancer susceptibility. But little is known about their association with risk and survival of breast cancer in Chinese population. The aim of this study is to examine whether cancer-related FTO polymorphisms are associated with risk and survival of breast cancer and BMI levels in controls in a Chinese population. We genotyped six FTO polymorphisms in a case-control study, including 537 breast cancer cases and 537 controls. FTO rs1477196 AA genotype had significant decreased breast cancer risk [odds ratio (OR) = 0.54, 95% confidence interval (CI): 0.34–0.86] compared to GG genotype, and this association was only found in women with BMI < 24 kg/m^2^ (OR = 0.41, 95% CI: 0.22–0.76); and rs16953002 AA genotype conferred significant increased breast cancer risk (OR = 1.80, 95% CI: 1.23–2.63) compared to GG genotype. Haplotype analysis showed that FTO TAC haplotype (rs9939609-rs1477196-rs1121980) had significant reduced breast cancer risk (OR = 0.76, 95% CI: 0.62–0.93) compared with TGC haplotype. But we failed to find any association between FTO polymorphisms and breast cancer survival. These findings suggest that variants in FTO gene may influence breast cancer susceptibility.

## 1. Introduction

Although breast cancer mortality rates have declined in recent years owing to increased awareness, early detection, and better treatment options, it is still the most common cancer and the leading cause of cancer-related death among females in the world [1]. In China, about 187,000 cases were diagnosed with breast cancer each year, of which approximately 48,000 died of this disease [2].

Potential risk factors, including age, reproductive factors, personal or family history of breast disease, genetic predisposition, and environmental factors, may be involved in the pathogenesis of breast cancer [3]. Early reports have described the clinical significance of genetic predisposition alleles in breast cancer development [4–6]. Fat mass and obesity-associated (FTO) gene plays a role in regulation of food intake and is originally identified as a susceptibility gene for obesity [7–9]. Obesity contributes to the development of a number of chronic diseases, such as metabolic syndrome, fatty liver, heart disease, and cancer [10–12]. It has been shown that the risk of cancer is 50% higher in obese women than those with normal weight, and there is growing scientific evidence that excess body weight contributes to the development of breast cancer [13, 14]. Although the underlying biological mechanisms by which body fatness becomes a risk for cancer development are not fully understood, recent studies have shown that a set of single nucleotide polymorphisms (SNP) in FTO gene may associate with cancer risk [15]. Till now, a number of FTO SNPs, such as rs1477196, rs11075995, rs17817449, rs11075995, and rs1121980, were reported to be associated with breast cancer risk in populations of different ethnic origins, but the results lack consistency and research was rare in Chinese population [16–20]. Besides, the association of FTO SNPs with survival of breast cancer has not been studied in Chinese population.

To explore the role of FTO polymorphisms in breast cancer development and progress, we performed a case-control study to assess the associations of six cancer-related FTO polymorphisms (rs9939609, rs1477196, rs6499640, rs16953002, rs11075995, and rs1121980) with breast cancer risk and prognosis and BMI levels in healthy controls in Chinese population.

## 2. Materials and Methods

### 2.1. Study Population

Newly pathologically confirmed female breast cancer cases were consecutively recruited between 2008 and 2014 from the Third Affiliated Hospital of Zhengzhou University. A total of 537 eligible breast cancer cases completed in-person interviews with response rate of 91.5%. At the same time, 537 healthy controls who frequency matched to cases by age (5-year-age groups) and without a history of cancer were randomly selected from the same hospital with response rate of 90.1%. At enrollment, all participants signed a written informed consent form and then were interviewed by trained interviewers using structured questionnaire, including demographic characteristics (age and education), reproductive variables (age at menarche, menopause status, and ever breastfeeding), medical information (histological grade, clinical stage, tumor size, estrogen receptor status, and progesterone receptor status), and family history of breast cancer. Height and weight were also measured to calculate body mass index (BMI). Fasting peripheral blood samples were also collected from each subject at enrollment. All patients were followed up regularly (every 6 months) until death or the end of follow-up. This study was approved by the Ethics Committee of the Third Affiliated Hospital of Zhengzhou University.

### 2.2. DNA Extraction and Genotyping

There are total 8 SNPs (rs9939609, rs17817449, rs8050136, rs1477196, rs6499640, rs16953002, rs11075995, and rs1121980) in FTO gene that reported to be associated with cancer risk [15]. After searching http://www.hapmap.org database, we found that rs9939609 tags rs17817449 and rs8050136 with *r* ^2^ > 0.95 in population of Chinese Han in Beijing (CHB). So we selected 6 SNPs (rs9939609, rs1477196, rs6499640, rs16953002, rs11075995, and rs1121980), all of which were located in the intron region of FTO gene, for genotyping with possible association with susceptibility and prognosis of breast cancer [15]. Genomic DNA was extracted from the buffy coats using genomic DNA purification kit (Promega, Madison, WI, USA). Genotyping for the selected SNPs was performed by ABI real-time PCR system using the TaqMan SNP genotyping assay. Polymerase chain reactions (PCR) were carried out by using standard PCR cycle conditions recommended by the manufacturer (an initial denaturation step at 95°C for 10 minutes, followed by 40 cycles of 95°C 15 seconds and annealing at 60°C for 1 minute). The PCR results were analyzed by use of the Detection System software. Finally, the quality and potential misclassification of the genotyping were assessed by reevaluating 10% of duplicate DNA samples (108 total samples) that are randomly selected from the whole study population. The concordance rate for the quality control samples was 100%.

### 2.3. Statistical Analysis

We used SAS software version 9.3 (SAS Institute, Inc.) for statistical analyses. Chi-square test and* t*-test were used to evaluate the case-control differences in the distribution of the selected characteristics, including age, education, age at menarche, menopause status, ever breastfeeding, family history of breast cancer, and BMI. Odd ratios (ORs) and 95% confidence intervals (CIs) of FTO SNPs and breast cancer risk were calculated by unconditional logistic regression models, using the most common genotype as the referent group. The linkage disequilibrium between loci in the FTO gene and the deviation of allele frequencies from Hardy-Weinberg equilibrium in healthy controls were assessed by HaploView version 4.2 [21, 22]. We used HAPSTAT 3.0 software to evaluate associations between haplotypes and cancer risk. Both univariate ANOVA and multivariate ANCOVA analyses were performed to determine the effects of the FTO polymorphisms on BMI levels in healthy controls. Effects of the different genotypes on breast cancer survival were evaluated by hazard ratios (HRs) and 95% CIs, using univariate and multivariate Cox regression analysis. A two-tailed *P* value of 0.05 was considered statistically significant.

## 3. Results

The distributions of selected characteristics of the study subjects are shown in Table 1. Cases and controls were evenly matched by age. Cases were more probably to have an earlier age at menarche and have higher BMI and lower education level and less likely to have been breastfeeding compared to healthy controls. No significant difference was found with respect to menopause status and family history of breast cancer between the two groups. The total positive expression rates of ER and PR among cases were 63.9% and 55.7%. The proportions of histologic grades I–III among cases were 27.9%, 42.3%, and 29.8%, and proportions of clinical stages 1-2 and 3-4 among cases were 76.2% and 23.8%, respectively. Besides, the percentages of patients with tumor size ≤ 2 cm and > 2 cm are 36.1% and 63.9%, respectively.

Frequencies of the 6 SNPs in FTO gene are shown in Table 2. Hardy-Weinberg equilibrium test of the selected SNPs showed no deviation among the controls (*P* > 0.05). Two SNPs in FTO gene were significantly associated with risk of breast cancer. Specifically, AA genotype of rs1477196 was associated with significant decreased risk of breast cancer (OR = 0.54, 95% CI: 0.34–0.86) compared with the GG genotype; subjects with the AA genotype of rs16953002 had significant increased cancer risk (OR = 1.80, 95% CI = 1.23–2.63) compared with those who carry the GG genotype. These associations were remained statistically significant after further adjustment for age, age at menarche, BMI, ever breastfeeding, and education (OR = 0.56, 95% CI = 0.34–0.90; OR = 1.77, 95% CI: 1.20–2.61). Further stratified analysis by BMI level (<24 kg/m^2^ and ≥24 kg/m^2^) showed that the effect of rs1477196 was statistically significant only in people with BMI < 24 kg/m^2^ (OR = 0.41, 95% CI: 0.22–0.76) (Table 3).

Haplotype analysis showed that rs9939609, rs1477196, and rs1121980 were in linkage disequilibrium (*D*′ = 1.0, *r* ^2^ = 0.07–0.78). The associations between FTO haplotypes and breast cancer risk were shown in Table 4. Carriers of the TAC haplotype had significant reduced risk of breast cancer (OR = 0.76, 95% CI: 0.62–0.93) relative to the TGC haplotype carriers.

Based on the data from follow-up interviews to October 2014, a total of 105 patients died of breast cancer. But we failed to find any association between FTO polymorphisms and breast cancer survival.

Finally, we investigated the associations between the FTO SNPs and BMI in healthy controls. Results showed that subjects carrying the mutant genotypes (G/A: 23.21 ± 3.25 kg/m^2^; A/A: 23.13 ± 3.30 kg/m^2^) of rs1477196 had lower BMI than those carrying the GG genotype (23.82 ± 3.60 kg/m^2^), but it did not reach statistical significance.

## 4. Discussion

FTO is an established obesity-susceptibility gene, and several loci in this gene have been reported to be associated with cancer risk. However, it has not been fully studied with regard to risk and survival of breast cancer in Chinese population. In this study, we found that two SNPs (rs1477196 and rs16953002) and TAC haplotype (rs9939609-rs1477196-rs1121980) in FTO gene were significantly associated with breast cancer risk. FTO gene located in chromosome 16q12.2. FTO protein is one homolog in the AlkB family proteins which also acts as a DNA-demethylase [23]. Mutations in FTO gene could lead to loss of protein function that may cause severe growth retardation, leanness, increased metabolic rate, and hyperphagia [24]. Previous genome-wide association study (GWAS) in 2007 has identified a set of susceptibility loci within the first intron of FTO gene that are obesity-related [25]. Also, a number of mutations in intron region of FTO gene were found to be associated with breast cancer risk in African-ancestry populations (rs17817449) [16], in a mixed ethnic population of Northwestern University (rs7206790, rs8047395, rs9939609, and rs1477196) [17], in ER-negative breast cancer of European ancestry population (rs11075995) [18], and in Chinese population (rs11075995) [20]. Although the exact functional significance of these SNPs has not been clarified, biological studies have suggested that the effect of the risk alleles can influence the methylation capability and at least in part be mediated through epigenetic alterations [26, 27]. Another study showed that the presence of the risk allele in intron 1 is associated with increased levels of the FTO transcript, suggesting that there may be* cis*-regulatory site that regulates FTO in intron 1 [28]. Thus, our findings are biologically plausible.

Rs1477196 located in intron 1 of FTO gene. A few studies have examined the effect of the rs1477196 variant on obesity and breast cancer risk. Rampersaud et al. [29] investigated the associations of physical activity, common FTO gene variants with BMI, and obesity in Old Order Amish (OOA) individuals, and the results showed that rs1477196 was significantly associated with BMI (*P* < 0.001), and this effect had significant interaction with physical activity (*P* = 0.004). Specifically, the rs1477196 C allele was associated with obesity in the low-activity group (OR, 1.58 for each risk allele; *P* = 0.001) but not in the high-activity group (*P* = 0.11). Another study done among early onset and severe obesity cases in a western Spain population also found that AA genotype of rs1477196 conferred decreased risk of obesity (OR = 0.41, 95% CI = 0.19–0.90), and a block containing the rs1477196/rs17817449/rs9939609 haplotype had an increased risk of obesity (GGA versus ATT: OR = 2.07, 95% CI = 1.41–3.04) [30]. A case-control study, including 354 breast cancer cases and 364 controls, conducted at Northwestern University in a mixed-race population found that compared to the common homozygote AA the rare homozygote GG of rs1477196 conferred 2.38-fold increased risk of breast cancer [17]. In our study, we found that rs1477196 AA genotype was associated with 0.54-fold decreased odds of breast cancer risk, and this association was found only in people with BMI < 24 kg/m^2^ after stratification by BMI levels.

Besides, TAC haplotype (rs9939609-rs1477196-rs1121980) had significant reduced breast cancer risk compared with TGC haplotype, which may be mainly driven by individual SNP rs1477196, which was in linkage disequilibrium with rs9939609 (*D*′ = 1.0) in our study. FTO rs9939609 has been reported to be associated with obesity in many other papers [31–33].

Rs16953002, located in 31kb from exon 9 and over 146kb from exon 8 of FTO, was once reported to be associated with melanoma in European population (per-allele OR for A = 1.16) [34], but till now, no other research has yet reported its role in obesity or breast cancer risk.

The essential strength of our study is the detailed review of cancer diagnosis (cases were histopathologically confirmed), which minimized the potential disease misclassification. However, limitations of our study cannot be ignored. First, potential selection bias of controls might occur because of the hospital-based study design. Second, Berkson's bias could not be ruled out because obesity is more frequent amongst hospitalized patients than that in the general population, which may underestimate the real association in our study. Third, the relatively small sample size may reduce the power to explore subgroups analysis. Fourth, lack of information on physical activity may have interaction with the identified mutations.

## 5. Conclusions

Overall, our results suggest that genetic variations in FTO gene may play roles in breast cancer pathogenesis in women. However, further researches are required to replicate our results in other populations and functional analyses are needed to elucidate the exact role of these genomic variations in the development of breast cancer.

## Figures and Tables

**Table 1:** Selected characteristics of cases and controls.

Characteristics	Cases (*n* = 537)	Controls (*n* = 537)	*P* value
Age (year)	47.9 ± 9.2	48.3 ± 8.9	0.50
Age at menarche	14.7 ± 1.7	14.9 ± 1.7	0.02
BMI (kg/m^2^)	24.0 ± 3.7	23.5 ± 3.4	0.007
Menopause	301 (56.1)	323 (60.1)	0.17
Family history of breast cancer	16 (3.0)	10 (1.9)	0.23
Ever breastfeeding	477 (88.8)	507 (94.4)	0.001
Education			
Junior middle school or below	258 (48.0)	204 (38.0)	0.002
Senior middle school	163 (30.4)	207 (38.5)	
College or above	116 (21.6)	126 (23.5)	
ER (*n*, %)			
Positive	343 (63.9)		
Negative	194 (36.1)		
PR (*n*, %)			
Positive	299 (55.7)		
Negative	238 (44.3)		
Histologic grade (*n*, %)			
I	150 (27.9)		
II	227 (42.3)		
III	160 (29.8)		
Clinical stage			
1-2	409 (76.2)		
3-4	128 (23.8)		
Tumor size (*n*, %)			
≤2	194 (36.1)		
>2	343 (63.9)		

**Table 2:** ORs and 95% CIs for breast cancer in relation to polymorphisms of FTO gene.

SNP	Genotypes	Cases, *n* (%)	Controls, *n* (%)	OR (95% CI)^†^	OR (95% CI)^‡^	^§^ *P* trend
rs9939609	TT	389 (72.6)	408 (76.0)	1.00	1.00	0.13
	TA	130 (24.2)	119 (22.1)	1.14 (0.86–1.52)	1.14 (0.85–1.52)	
	AA	17 (3.2)	10 (1.9)	1.78 (0.80–3.93)	1.69 (0.75–3.80)	
	AA + TA	147 (27.4)	129 (24.0)	1.19 (0.90–1.57)	1.18 (0.89–1.56)	

rs1477196	GG	272 (50.7)	231 (43.0)	1.00	1.00	0.004
	GA	231 (43.1)	254 (47.3)	0.77 (0.60–0.99)	0.79 (0.61–1.02)	
	AA	33 (6.2)	52 (9.7)	**0.54 (0.34–0.86)**	**0.56 (0.34–0.90)**	
	AA + GA	264 (49.2)	306 (57.0)	0.73 (0.58–0.93)	0.75 (0.59–0.96)	

rs6499640	GG	361 (67.5)	375 (69.8)	1.00	1.00	0.44
	GA	159 (29.7)	148 (27.6)	1.12 (0.85–1.46)	1.13 (0.86–1.48)	
	AA	15 (2.8)	14 (2.6)	1.10 (0.52–2.32)	1.11 (0.52–2.35)	
	AA + GA	174 (32.5)	162 (30.2)	1.11 (0.86–1.44)	1.13 (0.87–1.47)	

rs16953002	GG	217 (40.5)	250 (46.5)	1.00	1.00	0.005
	GA	232 (43.3)	231 (43.0)	1.16 (0.90–1.50)	1.13 (0.87–1.46)	
	AA	87 (16.2)	56 (10.4)	**1.80 (1.23–2.63)**	**1.77 (1.20–2.61)**	
	AA + GA	319 (59.5)	287 (53.5)	1.28 (1.01–1.64)	1.25 (0.98–1.60)	

rs11075995	TT	249 (46.5)	236 (44.0)	1.00	1.00	0.24
	TA	244 (45.5)	247 (46.0)	0.93 (0.73–1.20)	0.97 (0.75–1.25)	
	AA	43 (8.0)	54 (10.0)	0.75 (0.48–1.17)	0.80 (0.51–1.25)	
	AA + TA	287 (53.5)	301 (56.0)	0.90 (0.71–1.15)	0.94 (0.73–1.20)	

rs1121980	GG	360 (67.2)	377 (70.2)	1.00	1.00	0.19
	GA	154 (28.7)	145 (27.0)	1.11 (0.85–1.45)	1.11 (0.84–1.46)	
	AA	22 (4.1)	15 (2.8)	1.54 (0.79–3.02)	1.42 (0.71–2.82)	
	AA + GA	176 (32.8)	160 (29.8)	1.15 (0.89–1.49)	1.13 (0.87–1.48)	

^†^Adjusted for age.

^‡^Adjusted for age, age at menarche, BMI, ever breastfeeding, and education.

^§^ *P* trend is test of trend for the number of copies of the variant allele (0, 1 and 2).

**Table 3:** FTO rs1477196 and rs16953002 and breast cancer risk stratified by BMI levels.

SNP	Genotypes	BMI < 24		BMI ≥ 24	
		OR (95% CI)^†^	OR (95% CI)^‡^	OR (95% CI)^†^	OR (95% CI)^‡^
rs1477196	GG	1.00		1.00	
	GA	0.75 (0.54–1.05)	0.73 (0.52–1.02)	0.81 (0.56–1.18)	0.86 (0.58–1.27)
	AA	0.41 (0.22–0.76)	0.45 (0.24–0.85)	0.91 (0.42–1.98)	0.72 (0.34–1.52)
	*P*_interaction_				0.02

rs16953002	GG	1.00		1.00	
	GA	1.20 (0.85–1.69)	1.15 (0.81–1.63)	1.10 (0.74–1.63)	1.09 (0.73–1.63)
	AA	1.78 (1.07–2.96)	1.66 (0.99–2.79)	1.82 (1.01–3.27)	1.90 (1.05–3.46)
	*P*_interaction_				0.15

^†^Adjusted for age.

^‡^Adjusted for age, age at menarche, BMI, ever breastfeeding, and education.

**Table 4:** Association of haplotypes in the FTO gene with risk of breast cancer.

Haplotype^*∗*^	Cases, %	Controls, %	OR (95% CI)^†^	OR (95% CI)^‡^	*P* value
TGC	53.8	50.4	1.00	1.00	
TAC	27.7	33.3	0.76 (0.62–0.93)	0.78 (0.63–0.95)	0.009
AGT	15.3	12.9	1.09 (0.85–1.40)	1.08 (0.84–1.40)	0.51
TGT	3.2	3.3	0.91 (0.55–1.48)	0.87 (0.53–1.44)	0.69

^*∗*^In the order rs9939609, rs1477196, and rs1121980.

^†^Adjusted for age. ^‡^Adjusted for age, age at menarche, BMI, ever breastfeeding, and education.
